# Hypersensitivity Reactions Associated With Aesthetic Botulinum Toxin A Injections: A Case Series

**DOI:** 10.7759/cureus.95795

**Published:** 2025-10-31

**Authors:** Elizabeth Buchholz, Rungsima Wanitphakdeedecha, Santi Jaturawichanant, Kornvikka Pattanapran, Niamh Corduff

**Affiliations:** 1 Dermatology, RiverEnd Aesthetics, Newtown, AUS; 2 Dermatology, Mahidol University, Bangkok, THA; 3 Dermatology, B'est Clinic, Bangkok, THA; 4 Cosmetic Medicine, KVK Center Clinic, Bangkok, THA; 5 Plastic Surgery, RiverEnd Aesthetics, Newtown, AUS

**Keywords:** abobotulinumtoxina, botulinum toxin type a, complexing proteins, excipients, hypersensitivity reactions, incobotulinumtoxina, letibotulinumtoxina, low-immunogenicity formulations, onabotulinumtoxina, prabotulinumtoxina

## Abstract

Botulinum toxin type A (BoNT/A) is commonly used in aesthetic treatments for indications such as moderate-to-severe glabellar frown lines. While generally considered safe and effective at low doses, hypersensitivity reactions, though extremely rare, can occur and may be clinically significant. We present three cases suggestive of hypersensitivity reactions to BoNT/A and discuss their potential mechanisms and clinical implications. In the first case, a 44-year-old woman developed severe angioedema after resuming abobotulinumtoxinA injections following a 12-month pause. Subsequent intradermal tests with onabotulinumtoxinA and incobotulinumtoxinA produced localized reactions, suggesting hypersensitivity to a shared component across formulations, possibly the 150 kDa BoNT/A neurotoxin itself. In the second case, a 38-year-old woman experienced delayed erythema and swelling after letibotulinumtoxinA treatment but tolerated subsequent incobotulinumtoxinA injections, suggesting possible reactivity to complexing proteins or other non-core components rather than the neurotoxin. In the third case, a 50-year-old woman developed systemic symptoms and localized swelling after prabotulinumtoxinA administration, consistent with a delayed-type hypersensitivity reaction. These cases highlight the importance of considering patients’ medical histories, prior BoNT/A exposures, and potential risk factors for hypersensitivity. Although rare, such reactions can range from localized effects to systemic manifestations. Possible allergens include the core neurotoxin, complexing proteins, excipients, or manufacturing contaminants. We discuss the potential immunological mechanisms underlying these reactions and emphasize that highly purified, low-immunogenicity formulations may be preferable, particularly for patients with prior allergic histories or prolonged treatment courses. Overall, this case series underscores the importance of clinical vigilance, appropriate patient selection, and awareness of rare but meaningful hypersensitivity reactions to BoNT/A in aesthetic practice. Routine reporting and further study are essential to improving patient safety and refining the risk profile of these widely used treatments.

## Introduction

Botulinum toxin serotype A (BoNT/A) is commonly used in aesthetic treatments for indications such as moderate-to-severe glabellar frown lines [1]. The original regulatory approval in the United States specified a maximum accepted dose of 20 U for the BoNT/A formulation onabotulinumtoxinA (Botox®; Allergan Inc., Irvine, CA, USA). BoNT/A has a long-established record of safety and efficacy when administered at low doses [2]. Adverse events associated with BoNT/A are generally mild and self-limiting, while serious complications are rare and typically result from improper administration [3], excessive dosing, inaccurate injection sites, or toxin migration [4]. Allergic reactions to BoNT/A are uncommon but can range from mild, transient rashes to severe anaphylaxis [5]. Anaphylaxis presents as an acute onset of symptoms involving the skin and/or mucosal tissues, often manifesting as rashes, urticaria, erythema, and swelling [6]. It may also involve respiratory distress, hypotension, gastrointestinal symptoms, or even multiorgan dysfunction [7].

There are a few reported cases in the literature [2,4,6,8-14] in which BoNT/A has been confirmed to cause hypersensitivity reactions, and only three case reports describe anaphylaxis following BoNT/A administration. Of these, only two were considered likely to represent true anaphylaxis to BoNT/A. Hypersensitivity reactions to BoNT/A are likely underreported. It remains unclear whether trace amounts of BoNT/A entering systemic circulation trigger these reactions or whether they result from immune responses to the BoNT/A toxin itself, other formulation components (e.g., complexing proteins, excipients), diluents, or potential contaminants.

Normal immune systems can develop four types of hypersensitivity reactions [12,15]. Type I hypersensitivity develops rapidly when IgE antibodies trigger the release of histamine and other inflammatory mediators by basophils and mast cells [16], resulting in the characteristic manifestations of anaphylaxis. Type II hypersensitivity reactions occur primarily in autoimmune diseases, where IgG and IgM antibodies activate the complement system and mediate cytotoxic responses, as seen in bullous pemphigoid [17,18]. Type III hypersensitivity involves the accumulation of IgG, IgM, and IgA, which activate complement pathways and recruit polymorphonuclear leukocytes, leading to tissue injury [19]. The binding of IgG to circulating foreign antigens forms immune complexes that can precipitate autoimmune disorders such as vasculitis and systemic lupus erythematosus [20]. Type IV hypersensitivity reactions develop 48-72 hours after antigen exposure and are observed in allergic contact dermatitis [21], delayed drug reactions, erythema multiforme, and Stevens-Johnson syndrome [22]. Activation of T lymphocytes, eosinophils, and monocytes results in cytokine release and subsequent tissue injury [23].

Among patients already demonstrating hypersensitivity, further intradermal testing of the causative agent is neither feasible nor safe. In the absence of serological testing, clinicians should remain vigilant for signs and symptoms suggestive of hypersensitivity reactions. In such cases, it is essential to consider whether the response was induced by the diluent, excipients, contaminants, the BoNT/A molecule itself, or the associated non-toxic nonhemagglutinin and hemagglutinin proteins, collectively known as complexing proteins, that are part of the BoNT/A complex. Clinicians should also assess the temporal relationship between exposure and reaction, as well as the purity and immunogenicity of the BoNT/A formulations involved. Purity refers to the absence of additional complexing proteins, inactive neurotoxin, host-cell proteins, or manufacturing-related impurities beyond the core 150 kDa neurotoxin protein. While all BoNT/A formulations contain this core 150 kDa Hall strain toxin, only incobotulinumtoxinA (Xeomin®; Merz Pharmaceuticals GmbH, Frankfurt am Main, Germany), Medytox’s Coretox® (South Korea), relabotulinumtoxinA (Relfydess®; Galderma Pharmaceuticals, Fort Worth, TX, USA), and daxibotulinumtoxinA (Daxxify®; Revance Therapeutics, Nashville, TN, USA) are free of complexing proteins [24,25]. Formulations that contain complexing proteins include onabotulinumtoxinA (Botox®; Allergan Inc., Irvine, CA, USA), abobotulinumtoxinA (Dysport®; Ipsen Ltd, Berkshire, UK), prabotulinumtoxinA (Nabota®; Daewoong Pharmaceuticals, South Korea), Meditoxin® (Medytox Inc., South Korea), letibotulinumtoxinA (Botulax®; Hugel Inc., South Korea), and CBTX-A (Prosigne®; Lanzhou Institute of Biological Products, China) [26]. BoNT/A formulations also differ in excipient composition. Botox®, Nabota®, Meditoxin®, and Botulax® contain human serum albumin (HSA) and sodium chloride, while CBTX-A includes gelatin, dextran, and sucrose. Xeomin® contains sucrose and HSA, both of which are considered immunologically inert. Furthermore, studies have identified clostridial DNA contaminants in Botox® and flagellin in Dysport® [26]. To underscore the importance of clinical vigilance, we present three cases with clinical histories suggestive of hypersensitivity reactions to BoNT/A.

## Case presentation

Written informed consent for publication of their data was obtained from all patients.

Case 1 (Australia) 

Case History/Examination

In 2019, a 44-year-old woman developed an acute, severe angioedema reaction after resuming abobotulinumtoxinA injections in the periorbital region following a 12-month pause due to limited access to treatment. The patient had a history of an unspecified childhood reaction that was later diagnosed as a penicillin allergy in 2017 and confirmed in early adulthood by skin prick testing. Beginning in 2017, she received aesthetic injections of abobotulinumtoxinA diluted in preserved bacteriostatic normal saline and hyaluronic acid (HA) filler, without subsequent skin reactions. Additional “top-up” injections of abobotulinumtoxinA were administered primarily to the glabella, frontalis, platysma, and perioral areas at intervals of less than two weeks, continuing for approximately 12 months.

**Figure 1:**
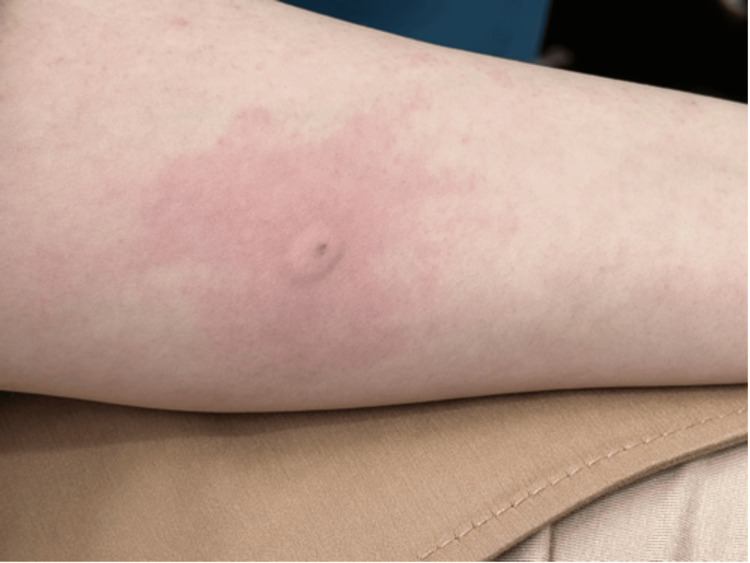
Localized, severe pruritus with a 1-cm wheal and a 5-cm flare at the injection site following an informal, in-clinic intradermal challenge injection on the forearm (Case 1).

**Figure 2:**
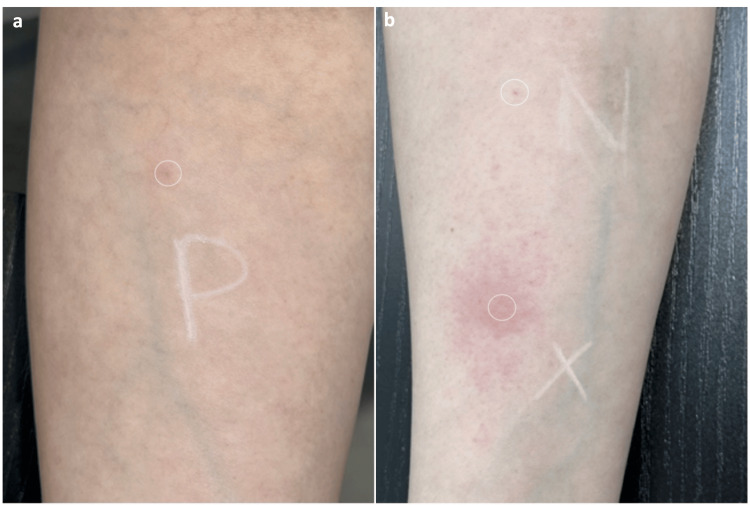
(a, b) No reaction was observed following saline injections, whereas a strong wheal and flare reaction occurred after intradermal injection of incobotulinumtoxinA (Case 1). P: preserved bacteriostatic saline injection containing benzyl alcohol (no reaction), N: normal non-preserved saline injection (0.9% NaCl; no reaction), X: incobotulinumtoxinA (5 units in 0.1 mL 0.9% NaCl) in non-preserved saline (wheal and flare reaction).

Top-up treatments were typically low-dose, except for masseter injections, which were given two to three times during this period. The acute angioedema reaction in 2019 required intravenous hydrocortisone, after which the patient improved rapidly, consistent with an immediate Type I hypersensitivity reaction. In 2020, an informal, in-clinic intradermal challenge on the forearm using a small dose of onabotulinumtoxinA produced a localized welt. In April 2023, another informal intradermal challenge with 5 U of incobotulinumtoxinA diluted in 0.1 mL preserved bacteriostatic normal saline resulted in an immediate hypersensitivity reaction characterized by severe pruritus, a 1-cm wheal, and a 5-cm flare at the injection site (Figure 1). In May 2023, testing was repeated with 0.1 mL plain normal saline, 0.1 mL preserved bacteriostatic saline, and 2.5 U of incobotulinumtoxinA diluted with plain saline. No reaction occurred with either saline, but a strong wheal and flare response occurred with incobotulinumtoxinA (Figures 2a-2b). Taken together, these findings indicate that all three BoNT/A formulations share a common allergen, most likely the 150 kDa BoNT/A toxin itself, which may explain the patient’s reaction to all formulations. Her documented penicillin allergy and prior positive skin prick testing further suggest a predisposition to allergic responses.

Case 2 (Thailand)

Case History/Examination

**Figure 3:**
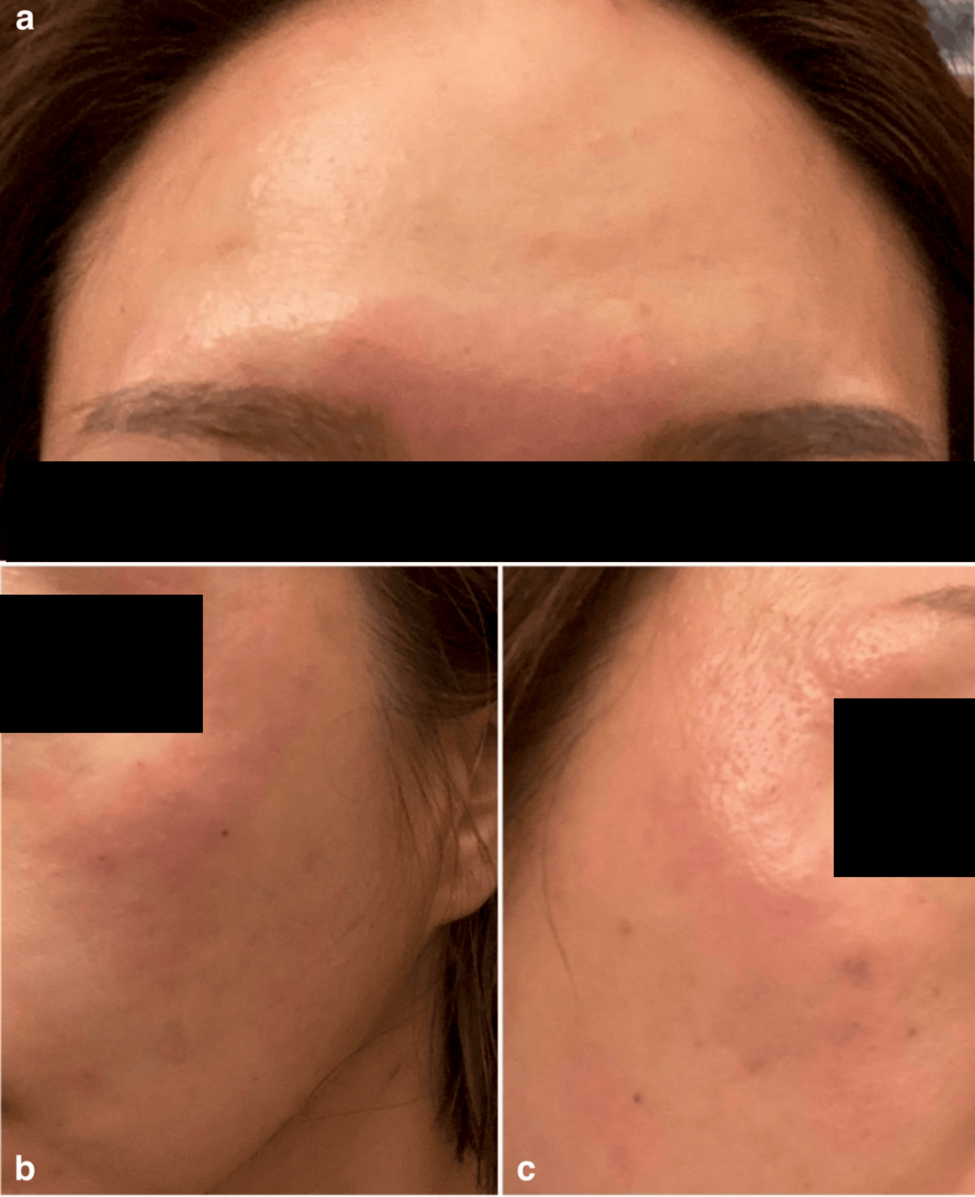
(a-c) Injection site erythema and swelling (left and center), which resolved completely three days after antihistamine treatment (Case 2).

A 38-year-old woman with no known allergies received treatment with an unspecified BoNT/A formulation in 2020, resulting in a good clinical response and no adverse effects. Twelve months later, she underwent injections of HA filler (Restylane; Galderma, Lausanne, Switzerland) into the bilateral tear troughs and letibotulinumtoxinA (diluted in normal saline; dose unknown) into the glabellar and crow’s feet regions. Approximately eight hours post-treatment, erythema and swelling developed at the BoNT/A injection sites, while the tear troughs treated with HA filler remained unaffected (Figure 3). The symptoms resolved completely within three days of antihistamine therapy. One month later, the patient received incobotulinumtoxinA injections to the upper face and masseters without recurrence of erythema or swelling. This presentation is suggestive of either an atypical Type I or a Type III hypersensitivity reaction, given the eight-hour delay between injection and symptom onset. The resolution with antihistamines supports an allergic mechanism, possibly related to BoNT/A or a contaminant. Because the patient tolerated subsequent incobotulinumtoxinA injections without incident, the 150 kDa BoNT/A molecule itself is unlikely to have been the allergen, implicating instead potential contaminants or complexing proteins as the trigger. A reaction to the HA filler or to topical agents used during the procedure cannot, however, be fully excluded.

Case 3 (Thailand)

Case History/Examination

**Figure 4:**
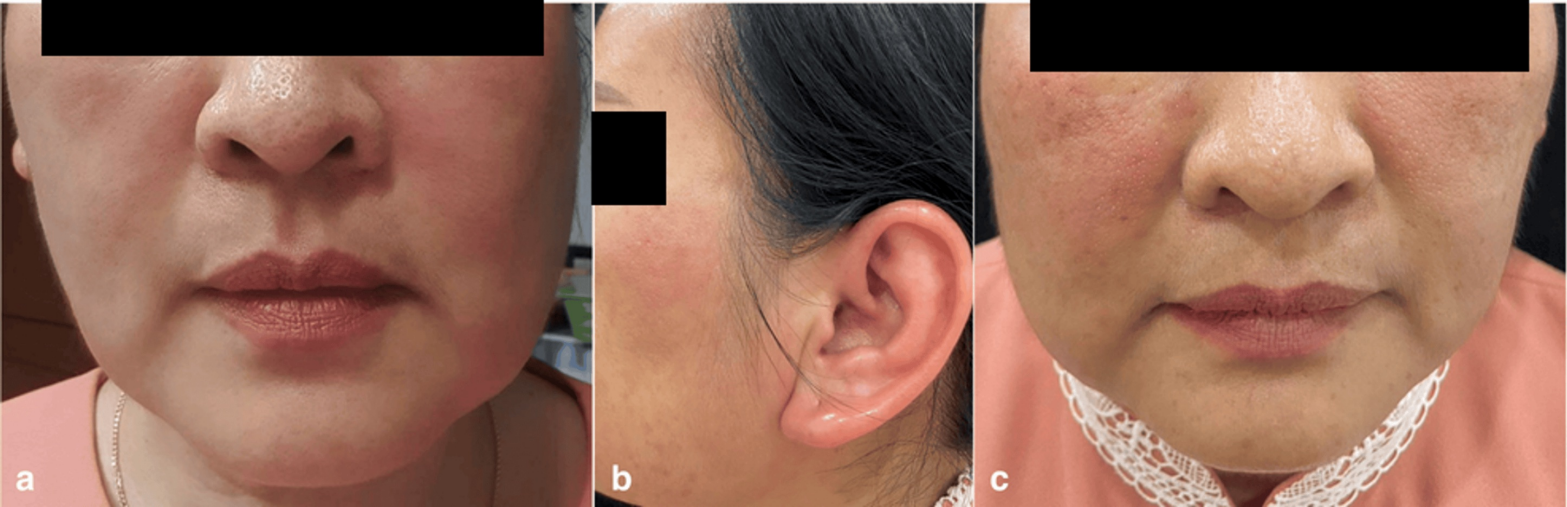
(a-c) Mild swelling and erythema of the cheeks, periorbital areas, and left ear helix (left and center), which subsided six hours after administration of oral prednisolone and antihistamines (right) (Case 3).

A 50-year-old woman with a history of allergic rhinitis but no known medication allergies had been receiving annual masseter injections with 50 U of prabotulinumtoxinA (diluted in normal saline) since 2015. In 2023, she additionally received 34 U of prabotulinumtoxinA in the upper face. The following morning, the patient reported feeling unwell, with low-grade fever, chills, and mild swelling and erythema of the cheeks and periorbital areas. Over the next four days, the erythema and swelling worsened and extended to the left ear helix. She also reported severe pruritus, myalgias, and a sensation of heat, although her temperature was not recorded. Four days after the BoNT/A injections, oral prednisolone and antihistamines were administered, and her symptoms began to subside within six hours (Figure 4). Given the delayed onset of symptoms, an IgE-mediated immediate-type reaction was unlikely. The clinical presentation was most consistent with a Type IV delayed-type hypersensitivity reaction, supported by the rapid response to glucocorticoid and antihistamine therapy. Differential diagnoses included photosensitivity and allergic contact dermatitis.

## Discussion

BoNT/A-related hypersensitivity is not well studied or documented due to its extreme rarity, low incidence rates, and a lack of published guidelines on how to diagnose or treat these reactions [12]. Our cases underscore the need for clinicians to consider patients’ past medical histories, allergies, and previous BoNT/A treatments (doses and formulations). In Case 1, it is possible that the initial treatment with abobotulinumtoxinA “primed” the patient’s immune system. The pharmacologically unnecessary bacterial components (hemagglutinins, flagellin) and/or potential unidentified contaminants in abobotulinumtoxinA injections may have served as adjuvants and activated an immune response to abobotulinumtoxinA, onabotulinumtoxinA, and incobotulinumtoxinA, the three most well-established aesthetic BoNT/A treatments with the longest history of commercial use in medical aesthetics. AbobotulinumtoxinA has the highest incidence of immunogenicity and is likely the most immunogenic [27]. HSA, lactose, normal saline, and sucrose, the excipients used in these preparations, are of a non-immunogenic nature, making them unlikely to elicit immune reactions [6]. It is likely that repeated injections of BoNT/A within short treatment intervals further increased the patient’s risk of developing an immune response. While Case 1 is more suggestive of a Type I hypersensitivity reaction, Cases 2 and 3 suggest other types. Case 2 aligns more with either an atypical Type I hypersensitivity reaction or a Type III reaction, which has been suspected in a previous case report [28]. Case 3 suggests a Type IV hypersensitivity reaction. We cannot say with certainty whether these are true hypersensitivity reactions as significant information is unavailable; formulation lot numbers and expiry dates, exact doses and injection dates, diluents, whether cleansing agents were used on the skin prior to treatment, the types of needles and gloves used, and any topical aftercare treatments. Intradermal injection for Case 1 was not performed in a formal allergy testing center. We cannot systematically exclude other diagnoses; however, the histories do suggest types of hypersensitivity reactions. Clinicians need to seek clarity from their patients or their previous healthcare providers regarding any prior reactions experienced. Hypersensitivity can be immediate or delayed, due directly to the BoNT/A molecule, a component in the formulation inherent to the product’s manufacturing processes, or contaminants unintentionally introduced subsequently during its handling and injection. As a case series on temporary and delayed hypersensitivity reactions to BoNT/A after COVID-19 vaccinations showed [29], the possible post-immunization immunogenic effects of BoNT/A were only established through the reporting and study of such rare events. 

Early reports [4] of hypersensitivity reactions after onabotulinumtoxinA injections include a patient being treated for unwanted mentalis muscle contractions who developed a persistent, localized rash at the injection sites, and a second patient receiving BoNT/A for lower leg dystonia who developed severe localized swelling. At the time, the product information for onabotulinumtoxinA mentioned cases of diffuse, localized swelling, and skin rashes [4]. Contemporary BoNT/A formulations use different genotypes or claim less or no immunogenicity [30], but there are multiple more recent examples of hypersensitivity reactions still occurring. After repeated onabotulinumtoxinA (20 U) injections for forehead and glabella lines, one patient with a prior history of allergic rhinitis developed swollen eyelids, severe localized itching, and erythema at the injection site [13]. Intradermal testing was positive, indicating delayed hypersensitivity due to Type IV, T-cell-mediated reactions, and confirming the potential for systemic hypersensitivity with onabotulinumtoxinA. In another patient treated for hemifacial spasms, initially with onabotulinumtoxinA and subsequently with a generic BoNT/A, periorbital and nasolabial edema developed an hour after administration [5]. A further patient developed injection site urticarial plaques 20 minutes after receiving Chinese BoNT/A (CBTX-A; Prosigne) [2], and intradermal testing produced plaques after 10 minutes, all of which resolved with systemic corticosteroids and antihistamines. Another case of a BoNT-related adverse event (AE) was induced by the intramuscular injection of BoNT/A into the masseter muscle of a patient with a prior history of AE-free treatment using the same formulation and treatment area [10]. Angio-edema and anaphylaxis developed quickly and required intervention with intramuscular epinephrine. Total serum IgE was subsequently found to be elevated in this patient, and intradermal testing indicated that the anaphylaxis was caused by BoNT/A. Therefore, while hypersensitivity reactions to BoNT/A are exceedingly rare, IgE-induced reactions can occur [5]. These reactions can also be induced by other toxin formulation components, such as gelatin in CBTX-A. Taken together, these incidents underscore the need for clinicians to be aware of the potential risks of excipients or unknown contaminants stimulating a hypersensitivity reaction, as extreme reactions such as anaphylaxis can be potentially life-threatening.

When administered by experienced injectors, BoNT/A treatments have a low risk of complications and are relatively safe [12], with allergic or hypersensitivity reactions being very rare [13]. AEs related to BoNT/A are largely mild and transient, and mild allergic reactions such as skin rashes are resolved mainly with antihistamines and steroids. However, severe and persistent complications have been reported, including anaphylaxis and granulomas [12]. It has not been possible to definitively attribute the complications to either the neurotoxin, its excipients, or potential contamination introduced during reconstitution [4,11,31]. Our real-world, retrospective, observational case series was also not a randomized clinical trial, and we could not systematically exclude potential contributing factors such as benzyl-alcohol in preserved saline, chlorhexidine/isopropyl preparations, latex, syringe lubricant, or unrelated dermatoses.

A systematic review [12] found that hypersensitivity manifested predominantly as erythema and pruritus at injection sites in the masseter, forehead, eyebrows, and orbicularis oculi, between 5 minutes and 36 hours post-injection, with patients also showing negative intradermal testing results. BoNT/A-related hypersensitivity reactions are mainly Type I [2], although Type IV reactions have been documented [10,13]. Botulinum toxin formulations contain components with the potential to elicit hypersensitivity, including the neurotoxin protein itself and non-toxin components, including excipients such as gelatin [10,13]. Although commonly used in pharmaceutical preparations like vaccines, gelatin can cause mast cell degranulation and, thus, severe adverse reactions [32,33]. As the core neurotoxin molecule is produced by *Clostridium botulinum *as a complex with different bacterial proteins (e.g., hemagglutinins and non-toxic non-hemagglutinins), these complexing proteins can potentially induce immune reactions and thus increase the risk of allergies. CBTX-A, which has gelatin as an excipient, was also linked to several adverse reactions [2,8] occurring between 24 and 48 hours post-injection and manifesting as injection site erythema and edema. Therefore, regardless of formulation, caution is warranted [34] when less highly purified toxin formulations containing immunogenic components are used repeatedly or over long durations.

IgE-mediated hypersensitivity responses can occur to all components of BoNT/A formulations, including the BoNT/A toxin itself [35]. The BoNT/A toxin, BTXA (Hengli, Lanzhou Institute of Biological Products, China), elicited an acute hypersensitive reaction of erythema in a site other than the injected area [36] in three patients. Erythema is a consequence of Type I hypersensitivity involving IgE, leading to mast cell degranulation with the production of inflammatory mediators such as histamine [16]. As noted by the investigators in this report, such hypersensitivity reactions should not be ignored or taken lightly, given that they may result in more serious consequences. In instances of potential IgE-mediated responses, clinicians must evaluate the type of BoNT/A administered, assess whether normal or preserved saline was used for dilution, and investigate any potential contaminants in the syringes or needles used. Strict hand hygiene and use of gloves must be ensured when diluting BoNT/A, and the cap must be sterilized with alcohol before being pierced with a clean needle. Finally, clinicians should consider any topical agents applied to the skin before or after treatment in the case of an allergic reaction. 

Low doses of BoNT/A are generally used in aesthetic procedures [37], and with some newer formulations containing lower non-BoNT/A protein loads than older formulations, the rates of antibody-induced immune reactions have also declined [2]. Using low doses and extended treatment intervals minimizes exposure to factors that enhance the potential for antibody formation. Careful selection of toxin formulations can also potentially mitigate hypersensitivity risks. IncobotulinumtoxinA has the lowest immunogenicity [38-41] among the established BoNT/A formulations. Two patients with cervical dystonia [14], who were subsequently switched to incobotulinumtoxinA, were initially administered repeated treatments with abobotulinumtoxinA and/or onabotulinumtoxinA over a long duration. Systemic hypersensitivity reactions (generalized skin rash, throat constriction, tachycardia, and fever) were reported with abobotulinumtoxinA and onabotulinumtoxinA but did not recur following the switch; therefore, incobotulinumtoxinA was continued with satisfactory results in both individuals. Additionally, HSA and sucrose in incobotulinumtoxinA formulations are both immunologically inert and have a long history of use in pharmacological preparations. IncobotulinumtoxinA itself has very high specific bioactivity, and its formulation is devoid of immunogenic inactive neurotoxins [42].

## Conclusions

BoNT/A hypersensitivity is rare, and clinicians may regard the associated harm as minimal, inconsequential, or not reportable. However, the potential impact and cost to the patient are neither insignificant nor ignorable. Although testing patients suspected of hypersensitivity may be impractical or impossible, such events should still be reported. Our cases demonstrate that hypersensitivity reactions can occur even with low-dose aesthetic treatments and may be precipitated by multiple factors, including contaminants. Clinicians should remain aware of the rare but potential risk of hypersensitivity, particularly in patients receiving long-term treatment. While hypersensitivity reactions to BoNT/A are uncommon, their clinical and personal consequences can be substantial. Careful history-taking, thorough documentation, and consistent pharmacovigilance reporting are crucial for enhancing recognition and understanding of these events. Risk mitigation strategies should include using the lowest effective dose, maintaining adequate intervals between treatments, and adhering to meticulous aseptic technique. Where hypersensitivity is suspected, early involvement of an allergist can support diagnosis and guide future management. Ongoing vigilance will help balance patient safety with the continued therapeutic and aesthetic benefits of botulinum toxin treatment.
